# RCSS: A Real-Time On-Demand Charging Scheduling Scheme for Wireless Rechargeable Sensor Networks

**DOI:** 10.3390/s18051601

**Published:** 2018-05-17

**Authors:** Ping Zhong, Yiwen Zhang, Shuaihua Ma, Xiaoyan Kui, Jianliang Gao

**Affiliations:** School of Information Science and Engineering, Central South University, Changsha 410083, China; ping.zhong@csu.edu.cn (P.Z.); YiWenZhang@csu.edu.cn (Y.Z.); shuaihuam@126.com (S.M.); xykui@csu.edu.cn (X.K.)

**Keywords:** adaptive charging threshold, energy consumption rate, on-demand charging scheduling, wireless rechargeable sensor networks (WRSNs)

## Abstract

With the emergence of edge computing, a large number of devices such as sensor nodes have been deployed in the edge network to sense and process data. However, how to provide real-time on-demand energy for these edge devices is a new challenge issue of edge networks. In real-world applications of edge computing, sensor nodes usually have different task burdens due to the environmental impact, which results in a dynamic change of the energy consumption rate at different nodes. Therefore, the traditional periodical charging mode cannot meet the nodes charging demand that have dynamic energy consumption. In this paper, we propose a real-time on-demand charging scheduling scheme (RCSS) under the condition of limited mobile charger capacity. In the process of building the charging path, RCSS adequately considers the dynamic energy consumption of different node, and puts forward the next node selection algorithm. At the same time, a method to determine the feasibility of charging circuit is also proposed to ensure the charging efficiency. During the charging process, RCSS is based on adaptive charging threshold to reduce node mortality. Compared with existing approaches, the proposed RCSS achieves better performance in the number of survival nodes, the average service time and charging efficiency.

## 1. Introduction

As a typical edge network, wireless rechargeable sensor network (WRSN) has been widely used in many different fields, such as military surveillance, disaster warning, medical system and so on [1,2,3]. The sensor nodes are powered by batteries, which will lead to energy limitations and high costs of frequent battery replacement. Therefore, how to prolong the service time of sensor nodes becomes a hot issue [4,5]. Current studies propose a variety of methods to prolong node lifetime and alleviate node energy restriction problem [6,7,8,9]. In these studies, dynamically adjusting the time duration of a node to stay active in one data collection period, i.e., duty cycle, is an efficient strategy to save energy and prolong the lifetime of network [2]. G. J. Han et al. [3] put forward an algorithm for improving the charging efficiency of the mobile chargers (MCs) under considering the relationship of the movement energy consumption of MCs and their energy transfer to the nodes in wireless sensor networks (WSNs). The energy crisis and carbon emissions have become critical concerns universally [10]. Under these circumstances, energy harvesting (EH) technology is especially concerned by the current researchers [2]. EH technology means that the nodes absorb solar energy, wind energy from the environment, and convert them into its own electrical energy to maintain its normal work. C. Wang et al. [4] proposed a hybrid framework that combines the two technologies, namely, cluster heads are equipped with solar panels to scavenge solar energy and the rest of nodes are powered by wireless charging. The simulation results demonstrated that the hybrid framework could reduce battery depletion by 20% and save vehicles’ moving cost by 25% compared to previous works. In [5], the authors proposed a data sensor resource allocation algorithm named DSRA to allocate the transmission time, power and channels so that the energy consumption of the data sensors was minimized. Zhang D et al. [6] formulated a maximization of minimum value problem which could be solved efficiently by utilizing its linear structure to achieve the optimal power control of each relay in EH wireless sensor network (EH-WSN). The previous methods can extend the lifetime of WSNs to a certain extent. However, due to the uncertainty of the environmental energy factors, the performance of the network using previous EH technology is unstable.

Fortunately, the wireless energy transmission (WET) technology sprang up as an alternative way to replenish the sensor node energy [7]. WET refers to the fact that there are one or more mobile charging vehicles equipped with a high energy capacity and providing energy for nodes in the network [8,9]. Comparing to EH technology, WET provides a more stable energy supply by the controllable charging power and mobile charger (MC). Therefore, WET is an emerging technology to enable the WRSNs perpetual operation. A mobile charging vehicle is an important component in WRSNs that using WET. The MC is responsible for providing the energy supply for the rechargeable sensor nodes. In [9,11,12,13,14], single to single charging mode is used to replenish the energy of nodes with predefined charging path. In order to increase the number of charging nodes at the same time, the one-to-many charging mode has been used in [15,16,17,18]. This charging mode means that the MC moves to a specific optimal location and charge all of nodes around its location simultaneously. However, the predefined charging path scheme is only suitable for static energy consumption networks, where the charging duration and the charging sequence in each charging cycle is always same. In practice, the amount of data collected by different nodes in different environments is distinct, which will cause the different energy consumption on each node. It is a process of dynamic change. Thus, it is necessary to design a scheduling method with adequately considering the dynamic energy consumption of different nodes, which is not fully considered in the existing literature. In this paper, we take the dynamic energy consumption of sensor nodes and the distance to mobile charging vehicle into consideration and propose an on-demand based real-time charging scheduling scheme (RCSS). Firstly, we propose a prediction model for the energy consumption rate of nodes. With the prediction model, the energy consumption rate of sensor nodes at each time period is effectively anticipated. Then, we design the charging scheme according to prediction model. The paper makes the following three main contributions.

- We propose a prediction model of energy consumption rate because the sensor nodes usually have different task burdens due to the environmental impact and the phenomenon results in a dynamic change of the energy consumption rate at different nodes. Based on the model and the distance between the MC and sensor nodes, the next charging node selection algorithm is presented. The algorithm can adapt to the real-time dynamic change of energy consumption in the network.
- Starting from the reality, the MC with limited energy is considered and we devise the feasible conditions of charging path to make sure the long-term operation of WRSNs. In addition, we optimize the charging threshold in charging process. The optimization of this parameter reduces the charging delay and the overall performance of the system has been significantly optimized.
- Through our extensive theoretical analysis and simulation study, we verify the accuracy of the model based on network simulation tool NS-3. The experiment has shown that, the proposed RCSS has a better performance in the number of survival nodes, the average service time and charging efficiency, which demonstrates the outstanding performance of the charging scheme.

The rest of this paper is organized as follows. Section 2 reviews the related work. The problem description and system model are presented in Section 3. In Section 4, we propose a real-time on-demand charging scheduling scheme in details. The simulation results are presented in Section 5. Section 6 is the conclusion of the paper.

## 2. Related Work

The sensor nodes are battery-powered and their energy is limited [19,20,21,22]. In order to solve the energy replenishment problem in WRSNs, many researchers have done a lot of research works, which can be divided into two types, periodical charging and on-demand charging. Most of existing studies adopt periodical charging scheme, which refers that the MC periodically traverses the nodes in the network and supplies energy to them. This method is generally used for the network where the node energy consumption rate is relatively stable. However, the energy consumption of the nodes in WRSNs shows dynamic variation characteristics because of the uncertainty of environmental change that causes the periodical charging scheme is not suitable for the network with complex environment changes. In contrast, charging on demand is more suitable for complex and changeable environments, which refers that the nodes send charging requests to the MC or the base station when the residual energy is less than the predefined energy threshold, and then the MC will charge the nodes or will be arranged by the base station to charge the nodes according to the charging requests received. In [23], the authors proposed a nearest first and recent rarest first methods to control the MC mobile charging path. In [24], C. Lin et al. proposed a game theoretical collaborative charging scheme, in which the MC could not only charge sensor nodes but also could add energy to other MC with low energy to ensure that as many nodes as possible to be charged. In [25], a greedy algorithm was proposed to full-charge the nodes with low energy level, which firstly ranked the nodes with energy requests in ascending order according to the amount of the residual energy, and then the lifetime of the first dead node is taken as the network lifetime. The mobile charging vehicle went to charge nodes by this ascending order. In [26,27,28,29], a queue model was used to investigate and analyze the charging process, and all of requests received by the mobile charging vehicle were stored in the buffer pool. The Nearest-Job-Next with Preemption (NJNP)NJNP method was proposed in [26,27], which used the most typical on-demand charging scheme. The MC always chose the nearest node in space from its buffer queue as the next charging node. The drawbacks of NJNP algorithm was it did not consider the residual energy of the nodes. It was assumed that the nodes near the MC have relatively sufficient amount of energy, and the energy of the node that was far away from MC is very low. Using NJNP, it would choose to charge the node closer to the MC, which would cause the death of nodes far away from MC. In [28], the authors proposed a Temporal and Distance Priority (TADP) charging scheduling algorithm named to quantify the priority of a charging task. The disadvantage of TADP was that no consideration is given to the impact of environmental changes on the energy consumption rate of nodes, which would lead to the different energy consumption of the nodes in each time interval. In practice, the MC with limited energy capacity will consume lots of energy for movement.

On-demanding charging plays important role in current edge computing [10,30,31,32,33,34,35,36,37]. In order to replenish energy from MC as many nodes as possible and prolong the lifetime of the entire network, some studies designed a feasible charging path for the MC. In literature [30,31,32], the maximum energy capacities of mobile charging vehicle and sensor nodes were given in advance as feasible conditions to judge whether the mobile charging vehicle could return to the service station before its energy depletion. The author in [33] used the target network life time to derive the feasible moving speed, charging rate and battery capacity of mobile charging vehicle. In [34], the maximum moving distance of mobile charging vehicle was given as a feasible condition to maximize the charging utility. The authors in [31] used the derived feasible charging scheme to select the primary nodes. J. X. Ren et al. [11] clustered the nodes in network according to their geographical position and the charging cycle was given in advance as a condition to charge the nodes. The condition was to make sure that the charging process of all nodes is successful. The goal in [35] was to minimize the charging cycle by calculating the optimal charging rate and optimal location of mobile charging vehicle. In brief, the summary of these related works above is listed in Table 1.

We find that most of the existing on-demand charging scheduling works decided the node charging order without considering the energy consumption rate of the nodes, which will cause that the nodes with high energy consumption rate are going to die because of the long waiting. This phenomenon will reduce the overall performance of the network. Meanwhile, some works assume that the energy capacity of the MC is infinite and it ignores the energy consumed for movement. These assumptions are not relevant in practical network applications. In this paper, we propose a real-time on-demand charging scheduling scheme, named RCSS to replenish the energy of sensor nodes and the feasible conditions are devised to decide whether the MC can guarantee the long-term operation of WRSN.

## 3. Problem Description and System Model

### 3.1. Problem Description

In practical networks, the energy consumption of each node varied with time due to the different location of the nodes and the influence of different environments [10,36,37]. In this paper, we propose a real-time on-demand charging scheduling scheme (RCSS) to charge the nodes that mainly aims at solving the following three problems.

- We consider the MC can only charge one node at the same time. When the MC receives multiple charging requests from nodes that need recharging, how to effectively arrange the charging order for MC to prevent nodes from dying due to energy exhaustion.
- To prevent other nodes from dying due to long waiting time, our method is to replenish proper amount of energy to the nodes instead of charging to its battery capacity. The problem is how to set the adaptive energy charging threshold.
- The service time and the number of charged nodes are limited due to MC limited energy capacity, thus we need to set up a proper path for MC to return to the service station for energy replenishment when its energy is pretty low.

By solving the above problems, we can maximize network lifetime and the survival nodes in each charging period.

### 3.2. System Model

**Figure 1:**
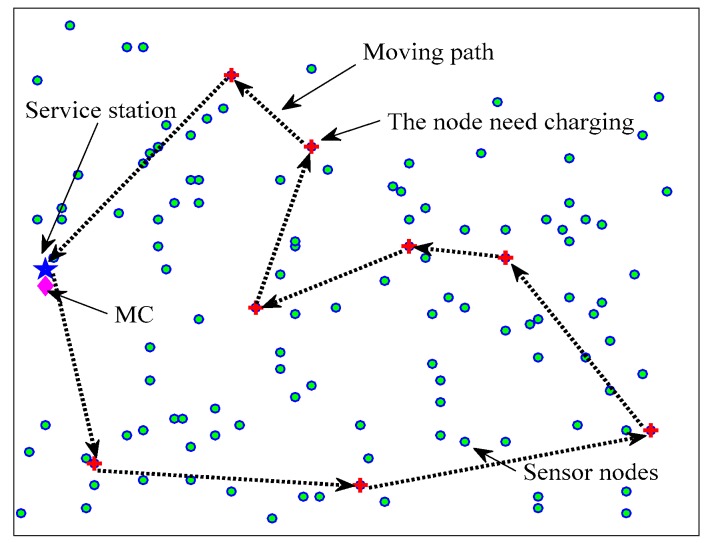
The topology model of network.

We assume that N rechargeable sensor nodes randomly distributed in a flat square area, as shown in Figure 1. The maximum battery capacity of the nodes is denoted as $E_{s}$. The sensor nodes are equipped with a wireless power receiver to harvest energy from MC. The MC with maximum capacity $E_{w}$ is a wireless energy transmission device and move on the flat ground at a constant speed *v*. It will return to the service station for energy supplement when the energy is insufficient.

The sensor nodes in the network detect the objects and send the perceived data to the base station. When the energy of the node is lower than predefined threshold $E_{th}$, it will send the energy request to MC for energy replenishment, which contains the node ID and the energy consumption rate. The MC will store all of the energy requests that have been received in its buffer pool, and then it uses the proposed next charging node selection algorithm (explained in detail in Section 4.1) to select one request from buffer pool and moves to the node to charge it with single to single charging mode. After charging the node, the MC selects another request using the same charging scheduling scheme from its buffer pool to serve.

## 4. Real-Time On-Demand Charging Scheduling Scheme

In this section, we will introduce the real-time on-demand charging scheduling scheme, RCSS, to solve the problems stated in Section 3.1. The scheme mainly consists of three parts, the next charging node selection, the feasible conditions for charging path and the adaptive energy charging threshold. We will discuss them in details below.

### 4.1. Next Charging Node Selection

Since the MC services the node with single to single charging mode, it must select one node to serve after receiving multiple charging requests. The request service order not only affects the charging required node, but also has a certain impact on other nodes that have not been served. For example, when the current serving node *i* with low energy consumption rate is very close to the MC, meanwhile there is a node *j* with higher energy consumption rate than node *i* in service pool and the distance from *j* to MC is relatively far, if we only consider the distance between the charging required node to MC, it may cause death of node *j* due to the rapidly energy consumption. If we only consider the energy consumption rate, the current serving node *i* with low energy consumption rate is very far from the MC, meanwhile there is a node *j* with higher energy consumption rate than node *i* in service pool is close to the MC, it will cause additional energy consumption because the MC moves backwards and forwards in network. Hence, we consider the energy consumption rate and the distance to MC as two important factors to select the next charging node. Firstly, we will introduce the prediction model of energy consumption rate for sensor nodes, and then we will present the process of next charging node selection.

#### 4.1.1. Prediction Model of Energy Consumption Rate for Sensor Nodes

When the sensor node detects that its energy is below a preset threshold $E_{th}$, it will send a charging request to the MC. The charging request contains the ID of the corresponding node and its energy consumption rate. After the MC has received the charging requests, it will estimate the energy consumption rate in the next period based on the current energy consumption rate of the node. It is assumed that the energy consumption rate of node *i* at time *t* is $R_{i}(t)$ and it becomes after Δ time. As shown in Equation (1), we give a prediction model of energy consumption in two states: charging and non-recharging. In Equation (1), α is a control factor (0 < *α* < 1) and $E_{i}(t)$ is the residual energy of node *i* at time *t*. When the node is in no-recharging state, it needs to compete with other requesting nodes for MC. Thus, we should utilize the nodes energy consumption rate of every Δ time to calculate the charging sequence. When the node is in charging state, it indicates that the charging sequence has been determined and it is very reasonable to use the current actual energy consumption rate instead of estimation. When the charging period is completed, reuse Equation (1) estimates the energy consumption rate of the charging nodes at the current moment until the next charging node is selected, and the MC performs the charging task. It is important to notice that our proposed energy consumption rate prediction model is time-dependent, which means that the energy change of any node can be predicted at different times and this is a real-time change process.

(1) $$R_{i}(t+\Delta )=\{\begin{array}{cc} (1-\alpha )R_{i}(t)+\alpha \frac{E_{i}(t-\Delta )-E_{i}(t)}{\Delta } & ,\;\mathrm{no}-\mathrm{recharging}\;\mathrm{state}\\ \frac{E_{i}(t-\Delta )-E_{i}(t)}{\Delta } & ,\;\mathrm{charging}\;\mathrm{state} \end{array}$$

#### 4.1.2. Next Charging Node Selection Algorithm

In general, the nodes with higher energy consumption rate have a higher charging frequency, meanwhile, the nodes that are closed to the MC wait for a shorter service time, and the MC also spends less energy on path movement. Therefore, we take both of the energy consumption rate and the distance to MC in space into consideration to decide the next charging node. As described in Algorithm 1, we mainly consider it in two aspects. On the one hand, we should select as close to the MC node as possible to reduce the additional energy consumption of MC moving back and forth between nodes. On the other hand, we should minimize the unnecessary delay to ensure that other nodes in service pool can be charged in time. When the MC needs to select the charging nodes, the energy consumption rate of the nodes in service pool is sorted from large to small and we can get the sort result $N_{r}$. The node *i* in $N_{r}$ is marked with the sort number as $N_{r}(i)$.The greater the energy consumption rate, that is the smaller the sorting number in $N_{r}$, indicates the more likely it is to become the next charging node. Then the distance from the nodes in service pool to the MC are sorted into a series of $N_{d}$.The node *i* in $N_{d}$ is marked with the sort number as $N_{d}(i)$.The shorter the distance from node to MC, that is the smaller the sorting number in $N_{d}$, implies the more likely it is to become a next charging node.

**Algorithm 1** Next Charging Node Selection AlgorithmInput: *n* (the number of charging nodes requested), $R_{i}(t)$, *D(i)* (the distance between MC and node *i*) Output: the minimum weight corresponding node While (*n*) The nodes that need to be charged are arranged in descending order according to the energy consumption rate $R_{i}(t)$. The sequence number of node I is marked as $N_{r}(i)$; The nodes that need to be charged are arranged in ascending order according to *D*(*i*). The sequence number of node *i* is marked as $N_{d}(i)$;  $P(i)=\beta N_{d}(i)+N_{r}(i)$;  If min *P*(*i*) is unique   The node corresponding to the min *P*(*i*) is selected as the next charging node;   Else    Select the node with minimum residual energy as the next charging node;   End if   *n* = *n* – 1; End while

The actual charging sequence of the node is determined according to the weight value of each node that we denote it as P(i). The value of P(i) is determined by the sorted results of the above two factors. Thus, we can get P(i) according to $P(i)=\beta N_{d}(i)+N_{r}(i)$. Considering that the distance in the actual scene is relatively large than energy consumption rate, we introduce a control factor β (0<β≤1) to neutralize the effect of distance. The value of *β* depends on the size of the network area and the number of nodes. When the network area size is large and the number of nodes is relatively small, the numerical difference between distance and energy consumption rate will be larger. Under this circumstance, *β* will take smaller value. The nodes with smaller value of P(i) have a higher priority to recharge the batteries. Thus, the MC selects the node with the minimum weight P(i) at each time as the next charging node. There is also a special case where multiple nodes have the same weight value. In this case, it is necessary to compare the residual energy of the nodes with the same weight value, and select the node with the least residual energy as the next charging node.

It is worth notice that the selection of each charging sequence only occurs when a node is charged or when a new energy request is reached. At the end of each charging node selection, only one charge request is selected. At the beginning of the next charging node selection, the previous order of the nodes should be cleared because the distance for the other nodes to MC change and the energy consumption rate vary at the same time. As we said above, the energy consumption rate prediction model is time-dependent and when a node is charged after determining the charge scheduling path of the MC, the distance from other charging nodes to the current MC location is variable, that is, the distance between all nodes and MC is also a real-time change process. Therefore, we can say that the process of next charging node selection is real-time and dynamic.

### 4.2. Feasible Conditions for Charging Path

Since the battery capacity of MC is limited, we need the mobile path of MC to be feasible so that it does not drain its energy before returning to the service station for additional power. Only in this way can the MC effectively charge the nodes that need to be charged in the network, accordingly, improving the network lifetime. We denote *T* as the charging cycle of charging path. $T_{i}$ is the arrival time of MC to node *i* and Tstay is the time that MC stays at the service station for energy replenishment. Assume there are *n* nodes that need charging in each charging tour, then the charging cycle *T* can be calculated as Equation (2), in which $T_{n}$ is the time when MC arrives at node *n*, $\tau_{n}$ is the charging time for node *n* and $v_{m}$ is the moving speed of MC. $D(n,0)/v_{m}$ is the time for MC returning from the last node *n* to service station. The time that MC arrives at node *i* can be calculated as Equation (3).

(2) $$T=T_{n}+\tau_{n}+\frac{D(n,0)}{v_{m}}+T_{stay}$$

(3) $$T_{i}=T_{i-1}+\tau_{i-1}+\frac{D(i-1,i)}{v_{m}}$$

Here we use D that indicates the whole closed length of the charging path, and then the Equation (2) can be represented by the time that MC spends on moving and charging nodes, that is Equation (4). The whole closed charging path length D can be calculated according to Equation (5).

(4) $$T=\frac{D}{v_{m}}+\sum_{i=1}^{n}\tau_{i}+T_{stay}$$

(5) $$D=\sum_{i=1}^{n}D(i-1,i)+D(n,0)$$

In order to ensure the normal operation of the nodes in each charging tour, the residual energy of nodes before charging should be satisfied with Equation (6), in which $R_{i}$ is the energy consumption rate of the next charging node *i*. Only in this way can the energy consumed by node *i* during the waiting time $\left(T_{i}-r_{i}\right)$ is not more than charging request threshold $E_{th}$. $r_{i}$ is the time when the energy charging request send. Otherwise the nodes will die because of energy exhaustion before charging.

(6) $$E_{th}-R_{i}\times (T_{i}-r_{i})>0$$

When the node is charging, it can communicate with each other at the same time. Therefore, the relation between the capacity $E_{s}$ of the nodes after charging and the charge energy satisfies the Equation (7). The charging rate of MC is *c* and *η* is the energy harvesting efficiency.

(7) $$E_{s}=E_{th}-R_{i}\times (T_{i}-r_{i}+\tau_{i})+\tau_{i}\times \eta \times c$$

In order to ensure the charging fairness among charging request nodes, each node can be charged only once in each path. In Equation (8), it is essential to ensure that the residual energy of the charging node in the path does not fall to the energy warning threshold. Plugging Equation (7) into Equation (8), we can get the Equation (9), which indicates that the energy replenished of node *i* is always more than energy consumed. In Equation (9), $\left(T-r_{i}\right)$ is denoted as the duration from sending energy request to the end of charging tour.

(8) $$E_{s}-R_{i}\times (T-T_{i}-\tau_{i})>E_{th}$$

(9) $$\tau_{i}\times c\times \eta >R_{i}\times (T-r_{i})$$

In a charging cycle, we extend the conclusion indicated in Equation (9) that the energy replenished by a single node is more than the energy consumed to the entire network. As shown in Equation (10), it means that all nodes are added more energy than they consume during the entire charging process.

(10) $$c\times \eta \times \sum_{i=1}^{n}\tau_{i}>R_{sum}\times (T-r_{i})$$

As we can see in Equation (4), the charging cycle is composed of total moving time and total charging time. Thus, we can get $\sum_{i=1}^{n}\tau_{i}<T-r_{i}$ in Equation (10). Accordingly, in order to make the Equation (10) still valid, we can obtain the Equation (11) as follows.

(11) $$c\times \eta >R_{sum}$$

Next, we will consider the conditions that MC maximum battery capacity $E_{w}$ needs to meet. In order to make sure that the MC complete charging tasks successfully and finally return to service station for energy replenishment, the maximum battery capacity of MC is not less than the sum of the total energy charged to nodes and the total energy consumed for movement. The condition that $E_{w}$ should satisfy is shown in Equation (12) in which $q_{m}$ is the energy consumed for moving per meter.

(12) $$E_{w}\geq c\times \sum_{i=1}^{n}\tau_{i}+D\times q_{m}$$

In summary, we get the feasible conditions for charging path. When and only when the nodes and MC are satisfied with Equations (11) and (12) respectively, we can say the charging path is feasible.

### 4.3. Real-Time On-Demand Charging Scheduling Scheme

In this section, we will introduce RCSS. First of all, we present an adaptive energy charging threshold to prevent the situation where other nodes cannot be charged in time due to long time for waiting, and then we will discuss the real-time on-demand charging scheduling algorithm in details.

#### 4.3.1. Adaptive Energy Charging Threshold

The adaptive energy charging threshold $E_{ad}$ indicates the node has sufficient energy to operate. The value of $E_{ad}$ is between the maximum battery capacity $E_{s}$ of node and the energy request threshold $E_{th}$. Once the residual energy reaches to $E_{ad}$, the charging node stops charging process, which means that it sacrifices its rest charging time for other nodes with extremely high energy crisis so that they can be charged before the energy is exhausted. We decide the adaptive energy threshold through the number of requests in the service pool and the number of nodes in network. The calculation of adaptive energy threshold is presented in Equation (13). The node stops charging when its energy reaches $E_{th}$ and other nodes with less residual energy can gain MC energy supply ahead of schedule, which allows more nodes to get charged per charging tour.

(13) $$E_{ad}=(E_{s}-E_{th})\times \frac{\sqrt{N-n}}{\sqrt{N}}+E_{th}$$

#### 4.3.2. Real-Time On-Demand Charging Scheduling Algorithm

In a practical network, MC mobile energy consumption is more than the energy consumed for charging sensor nodes. Therefore, we cannot ignore the energy consumed for movement of MC. After a certain period of time, the MC moves to the service station for energy supplement. It is necessary to set up a charging path for MC to return to the service station for energy replenishment before energy depletion.

We mark as $N_{0}$ the service station for convenience. The real-time on-demand charging scheduling algorithm is shown in Algorithm 2. From the line 6 in Algorithm 2, the MC selects the next charging node using the Algorithm 1. After the first charging node is found, MC creates a virtual closed path that returns the service station through the selected charging node, and then the feasible conditions for charging path is used to judge the feasibility of this virtual closed path. If the path is feasible and the residual energy of the node can maintain the node working properly until the MC reaching, the MC moves to the node and charges the node until its energy reaches $E_{ad}$. Otherwise, MC gives up the selected node and uses the Algorithm 1 again to select the next charging node. When the charging process of selected node is completed, the MC will repeat the next charging node selection algorithm and the path feasibility condition until the path is infeasible or there are no charging requests in service pool, and then the MC will directly go back to the service station for energy supplement. The establishment of the charging scheduling path is a real-time process. The charging request is dynamic during a charging cycle instead of designing ahead of time. Compared with the scheduling method of pre-set charging path, the real-time charging path is more suitable for the network with dynamic energy consumption.

**Algorithm 2** Real-Time On-Demand Charging Scheduling AlgorithmInput: *n* (the number of charging nodes requested), *N_i_* (the node ID, 0<i≤n) Output: charging path *X* Mark $N_{0}$ as service station While (n!=0) do MC starts from $N_{0}$; Select the first charging node *N_i_* using the Algorithm 1; Build a virtual closed charging path $X(N_{0},N_{i},N_{0})$; While (*X* is feasible && *n*) do If ($E_{th}-p_{i}\times T_{i}>0$)  Put the selected node into charging path, the MC moves to the selected node and charge it until its energy is $E_{ad}$;  MC updates its residual energy; Else  n−−;   If (n−−)    Select the next charging node $N_{k}$ with the Algorithm 1;    Update the virtual closed path $X(N_{0},N_{i},N_{k},N_{0})$;   End if  End if End while The MC returns to $N_{0}$ for energy replenishment; End while

### 4.4. Case Study

**Figure 2:**
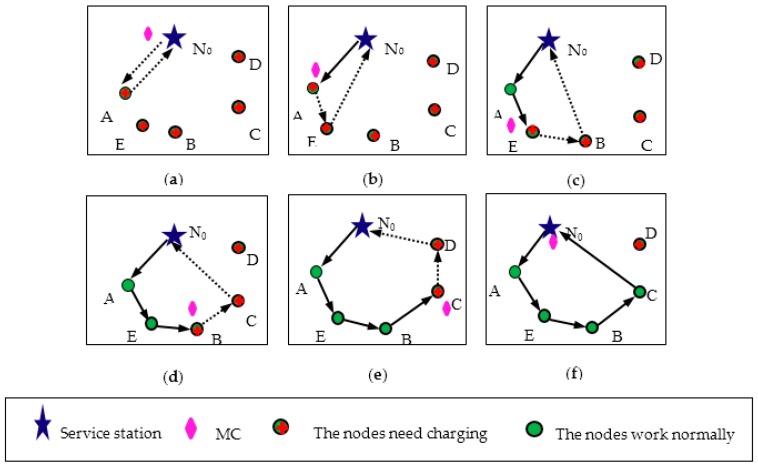
Charging path construction process. (**a**)–(**d**) The node with the smallest weight is chosen as the next charging node to build the virtual charging path successfully. (**e**) The fifth charging node selection and virtual path construction failure. (**f**) MC returns to the starting point for energy replenishment of and prepares for the next round of charging.

In order to better demonstrate the operation of our proposed on-demand charging scheduling algorithm, we will use an example to illustrate the specific operation process. Assume there are five nodes need to be charged and there is no other new energy request come. When the MC receives the five requests, the Algorithm 1 is used to select the node to be charged. The weight values P(i) of each node are obtained by sorting the energy consumption rate $N_{r}(i)$ of the five nodes and the distance $N_{d}(i)$ to the MC. As listed in Table 2a, we calculate the weight values of each node using the control factor β=1. The node A has the minimum weight value, so MC creates a virtual closed path through the node A and returns the service station, which is shown in Figure 2a using the dotted line. By determining the path feasibility and residual energy, the node A can be decided as the next charging node, thus the MC moves to node A and serve it until its residual energy obtaining $E_{ad}$. After the charging process has been completed, the energy consumption rate $N_{r}(i)$ of the remaining nodes and the distance $N_{d}(i)$ to the MC are re-sorted, and then the corresponding weight values are obtained, which is listed in Table 2b. By comparing the weight values, the node E with the minimum weight value is selected as the next charging node and the virtual closed path of the service station is returned through the node E, which is shown in Figure 2b. Through path feasibility determination and residual energy estimation, the node E can be determined as the next charging node. Repeat the “sort, select and judge” process, MC can complete the charging process for node B and node C, which is listed in Table 2c,d and Figure 2c,d. Finally, the virtual closed path is constructed for the node D, as shown in Figure 2e. According to the feasibility condition of the path, it is found that the path is infeasible, so MC is returned directly to the service station from node C, which is shown in Figure 2f.

It is important to note that the weight order of the five nodes is selected by four comparisons. Each movement of MC results in dynamic changes in the distance from each node MC, and the energy consumption of each node changes dynamically with time, hence, the mobile path construction of MC is a process of real-time change. For node D, The energy supplement will be carried out during the next charging process. This means sacrificing some nodes that cannot satisfy the path feasibility to satisfy more nodes and get MC services. From the global perspective, this method can ensure more nodes to survive. The nodes with high priority but unable to be scheduled into a feasible path of the MC will be first responds to the next round of charging.

## 5. Performance Evaluation

In this section, we evaluate the performance of our proposed on-demand charging scheduling scheme. We mainly compare with TADP [25]. TADP mainly considers the distance between nodes and MC and the remaining life of nodes, and MC will leave every time the nodes are full of electricity. Moreover, the results are compared with the earliest deadline first algorithm (EDF). The core idea of EDF is to select the nodes with the shortest remaining life to serve first. When using TADP, MC will choose a node with less remaining life and closer to the MC, however, it ignores such a situation that there are two nodes have the same distance to MC, if the node *i* has less remaining life and less energy consumption rate, and the other node *j* has relatively larger remaining life than node *i*. But the energy consumption rate of node *j* is far greater than node *i*. In this case, the node *j* is more important than the node *i* and MC should first serve node *j*, so the energy consumption rate is a more important metrics than the remaining energy. When using EDF, MC selects the node service with less remaining life and neglects the distance from the node to the MC, which will result in more energy spent on the MC. RCSS takes into account the energy consumption rate and distance to select the next charging nodes, and adopts adaptive threshold in charging process, which explains in theory that RCSS is better than TADP and EDF. Then we will test our theory by simulation experiments.

We accomplish the simulation with NS-3 simulator. The parameters for RF-based energy transfer system are based on the *TB-Powercast* sensor nodes. The parameter settings we used are listed in Table 3. In the experiment, the area size of the network we considered is relatively small. The numerical difference between the distance between nodes and energy consumption rate is relatively small, so we set the value of *β* to 0.8.

In this section, extensive simulations are conducted to prove the advantages of real-time on-demand charging scheduling algorithm by compared with TADP and EDF. Since we have considered that MC has a limited battery capacity, in order to evaluate the performance better, we consider that TADP and EDF also have limited energy source capacity in contrast experiments. We mainly evaluate the above three algorithms in terms of charging throughput, the number of survival nodes, average charging response time, average service time, and charging efficiency.

### 5.1. Charging Throughput

**Figure 3:**
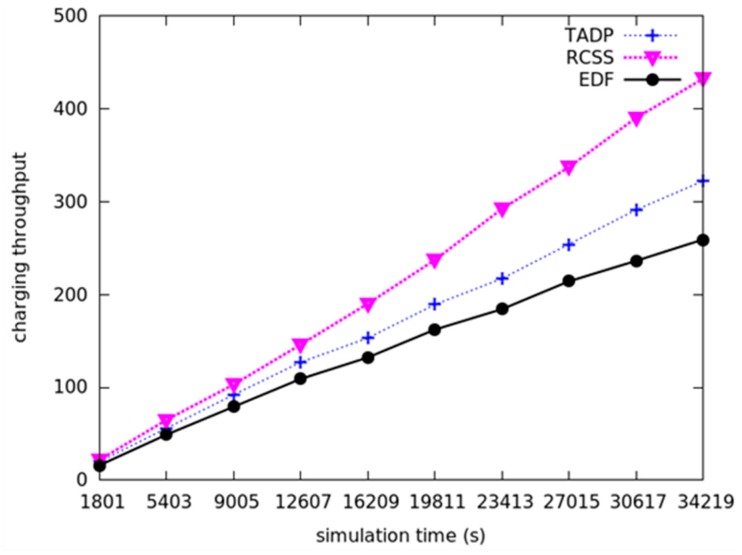
The comparison of charging throughput among RCSS, TADP and EDF.

Charging throughput is one of the important evaluation metrics of charging scheduling algorithms. The charging throughput is defined as the number of nodes that get charged successfully at each time period. The more nodes get charged means the less time for movement of MC. We assume that 100 nodes are randomly distributed in the network with size of 100 m × 100 m. As shown in Figure 3, when the simulation time continuously goes forward, the charging throughput increase monotonously, moreover, the charging throughput of our proposed algorithm (RCSS representation) is higher than TADP and EDF algorithms at each time period. From the figure, it seems that the three methods of charging throughput are the same in 1801 s in the experiment. In fact, the charging throughput in 1801 s of RCSS is 23, while the TADP and EDF are 20 and 17, respectively. It turns out that our proposed algorithm can make lots of nodes get charged before their deadlines, thereby effectively prolonging the lifetime of the entire network. There are two reasons for RCSS that has high charging throughput. One is that MC selects the best charging node by using path feasibility conditions when choosing the charging node, which means sacrificing some nodes with higher priority but not satisfying the path feasibility condition and more nodes with relatively low priority but the node that satisfies the path feasibility condition can replenish the energy in time. The other is that RCSS uses the adaptive charging threshold during the charging process, which can reduce the waiting time of the other nodes and achieve a higher charging throughput.

### 5.2. Average Service Time

**Figure 4:**
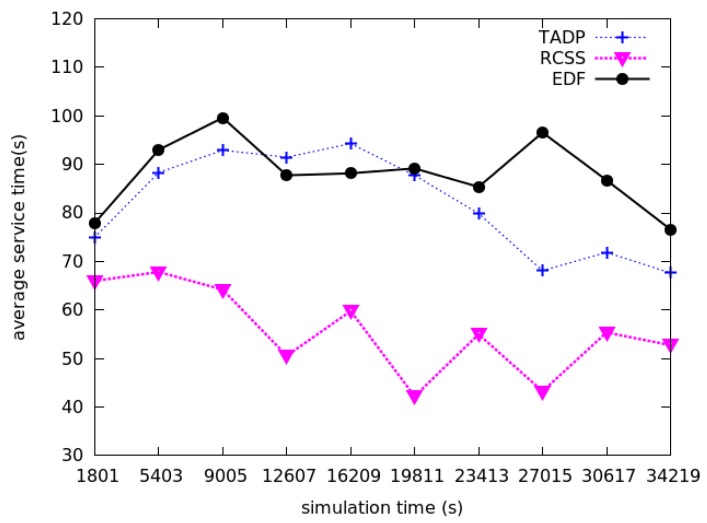
The comparison of average service time among RCSS, TADP and EDF.

Now we will compare the average service time among these three charging scheduling algorithms. We denote the average service time as the time period between the time when the request is responded by MC and the time when the requests get served completely by MC. The average service time can reflect the charging efficiency. The average service time is less, which means the waiting time of other nodes is shorter and the probability of death due to energy exhaustion is smaller. As we can see from Figure 4, the average service time used to charge nodes of our proposed algorithm is much lower than TADP and EDF algorithms at each time period assuming that there are 100 sensor nodes in the network with size of 100 m × 100 m. It is important to notice that RCSS uses adaptive threshold charging during the charging process, which means that the size of the charging threshold determines the duration of the charging. The increase in charging throughput, that is the increase in the number of nodes of the charge, does not mean that the average service duration decreases. The reason is that if the adaptive threshold is higher, the average service duration will increase. Therefore, the average service time will fluctuate. The average service time value under different algorithms is listed in Table 4. We can see that the average service time value of our proposed algorithm is almost half of the EDF based algorithm, which turns out that our real-time on-demand charging scheduling algorithm makes the waiting time of request shorter and more nodes get charged.

### 5.3. Average Charging Response Time

Then we will evaluate the average charging response time of requests, which is defined as the time period between the time the node sends the charging requests and the charging requests is responded. We also can regard it as the charging delay. The average charging response time reflects the waiting time before the request charging nodes get charged, moreover, it is also one of the important metric to evaluate the performance of charging scheduling algorithms. It is clear to see in Table 5, the average charging response time of RCSS is 1168.15 s, which is far less than TADP and EDF. It powerfully proves the effectiveness of our proposed algorithm. The main reason for this phenomenon is that the adaptive charging threshold is used in RCSS, while MC does not leave to serve the next node until the current node is full of energy in TADP and EDF, which will lead to the increase of the waiting time of other nodes.

### 5.4. The Number of Surviving Nodes

**Figure 5:**
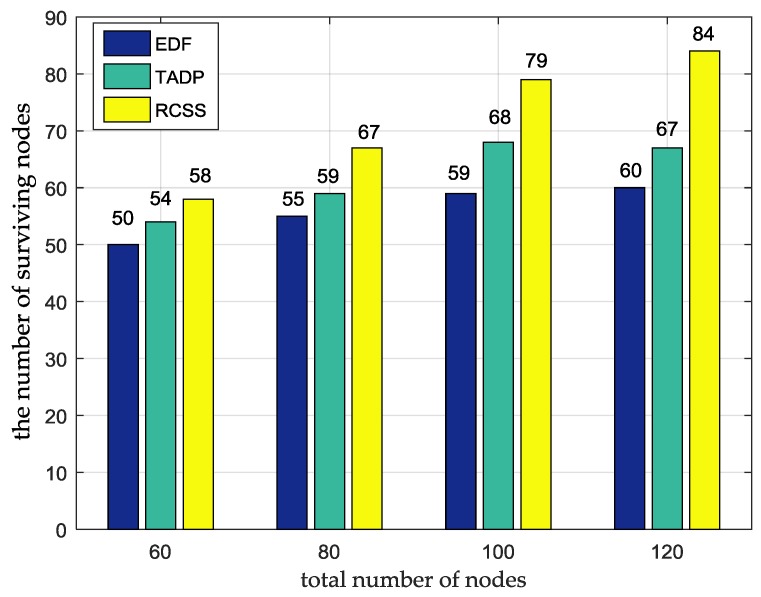
The comparison of the number of surviving nodes among RCSS, TADP and EDF.

We consider the nodes randomly distributed in the flat square area with size of 100 m×100 m, and the total number of nodes varies from 60 to 120. It is clear in Figure 5 that the number of surviving nodes using our proposed algorithm is always more than TADP and EDF algorithms no matter what the total number of nodes is. This is because we consider both the energy consumption rate and the distance into consideration in the next node selection algorithm, which are not done in TADP and EDF, and the adaptive charging threshold is used during the charging process, which ensures that more nodes can be recharged. Taking the total number as 120 as an example, RSCC survives 84 nodes in the experimental duration, while EDF and TADP are 60 and 67 respectively, which obviously shows that RCSS improves the survival rate of nodes. For better demonstrating the effectiveness of our proposed adaptive charging threshold, we conduct experiment without adaptive charging threshold. The experimental data are presented in Table 6, our proposed algorithm without adaptive charging threshold is also better than TADP and EDF algorithms. However, our proposed algorithm with adaptive charging threshold has four more nodes survived than our proposed algorithm without adaptive charging threshold and this proves that the necessity of the existence of adaptive charging threshold.

### 5.5. Charging Efficiency

**Figure 6:**
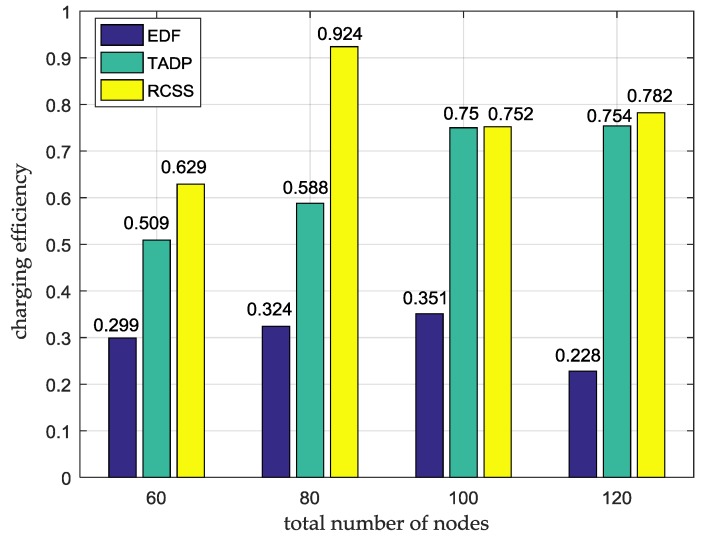
The comparison of charging efficiency among RCSS, TADP and EDF.

In the on-demand charging framework, the charging efficiency is an important evaluation metric of charging scheduling algorithm. The charging efficiency is defined as the ratio of the amount of energy charged to the amount of energy consumed for movement by MC in whole system. The higher the charging efficiency means the better the overall performance of the network. We also consider that the nodes are randomly distributed in a 100 m × 100 m flat square area with the total number of nodes varying from 60 to 120. Figure 6 shows the evaluation results on the charging efficiency under different total numbers of nodes. It can be clearly observed that, regardless of the total number of nodes, our algorithm is superior to the other two algorithms in charging efficiency, which is more obvious when the number of nodes is 80. This is because our algorithm takes the distance and energy consumption rate of two important factors into account. In addition, we set the path feasibility condition. The condition ensures that MC can smoothly return to the service station for energy supplement, instead of selecting far distance charging nodes to serve. Therefore, more energy is used to supplement nodes rather than MC mobile consumption. Taking the total number as 80 as an example, the charging efficiency of RSCC is 0.924, while the charging efficiency of EDF and TADP is 0.324 and 0.588 respectively, which clearly shows that MC makes more full use of energy under RCSS.

## 6. Conclusions

In this paper, we proposed a real-time on-demand charging scheduling scheme (RCSS) for WRSNs. In RCSS scheme, an efficient algorithm of the next charging node selection is proposed by taking the capability of mobile chargers into account, which is more practical for real-world edge computing applications. Then, in order to balance the delay of overall network nodes that need recharging, we propose an adaptive charging threshold to make the more nodes get charged in time. In RCSS scheme, the path selection of mobile charger is a dynamic process because the energy consumption rate and the location of the distance MC are changed in real time. Finally, extensive simulations have been conducted to evaluate the performance of our proposed RCSS, and the results showed that our algorithm is better than other two charging algorithms (TADP and EDF) in terms of the number of survival nodes, charging throughput, charging efficiency.

## Figures and Tables

**Table 1:** The summary of related works.

Literature	Real-Time Scheduling	Constraints of MC Energy	Adaptive Charging Threshold	Feasibility Guarantee
J. Wang et al. [23]	Yes	No	No	No
C. Lin et al. [24]	Yes	Yes	No	No
L. He et al. [26]	Yes	No	No	No
L. He et al. [27]	Yes	No	No	No
C. Hu et al. [30]	No	Yes	No	Yes
C. Lin et al. [28]	Yes	No	No	No
J. X. Ren et al. [11]	No	Yes	No	Yes
Y. Feng et al. [29]	Yes	No	No	Yes
C. Lin et al. [31]	No	Yes	No	Yes
G. Y. Jiang et al. [32]	No	Yes	No	Yes
X. Ye et al. [34]	No	No	No	Yes
X. Rao et al. [35]	No	No	No	Yes
Our paper	Yes	Yes	Yes	Yes

**Table:** (**a**)

	A	B	C	D	E
$N_{r}(i)$	1	3	4	5	2
$N_{d}(i)$	3	5	1	2	4
P(i)	4	8	5	7	6

**Table:** (**b**)

	B	C	D	E
$N_{r}(i)$	3	4	2	1
$N_{d}(i)$	1	3	4	2
P(i)	4	7	6	3

**Table:** (**c**)

	B	C	D
$N_{r}(i)$	2	1	3
$N_{d}(i)$	1	3	2
P(i)	3	4	5

**Table:** (**d**)

	C	D
$N_{r}(i)$	1	2
$N_{d}(i)$	1	2
P(i)	2	4

**Table 3:** Parameter settings.

Parameters	Value
Number of nodes	20–100
Operating voltage	3.6 V
Energy capacity of nodes	500 J
Energy capacity of the MC	50,000 J
Threshold for charging requests	225 J
Charging rate	5 w/s
Moving consumption	10 J/m
Mobile speed of MC	3 m/s
Simulation time	36,000 s
*β*	0.8

**Table 4:** The value of average service time.

Algorithm	Average Service Time
TADP	79.78 s
EDF	87.72 s
RCSS	55 s

**Table 5:** The value of average charging response time.

Algorithm	Average Charging Response Time
TADP	1923.46 s
EDF	2616.65 s
RCSS	1168.15 s

**Table 6:** The number of survival nodes under different algorithm.

Algorithm	Total Number of Nodes	The Number of Survival Nodes
TADP	100	59
EDF	100	68
RCSS	100	79
RCSS without adaptive charging threshold	100	75
